# Menopausal hormone therapy is associated with worse levels of Alzheimer's disease biomarkers in *APOE* ε4‐carrying women: An observational study

**DOI:** 10.1002/alz.14456

**Published:** 2025-01-09

**Authors:** Ainara Jauregi‐Zinkunegi, Carey E. Gleason, Barbara Bendlin, Ozioma Okonkwo, Bruce P. Hermann, Kaj Blennow, Henrik Zetterberg, Eef Hogervorst, Sterling C. Johnson, Rebecca Langhough, Kimberly D. Mueller, Davide Bruno

**Affiliations:** ^1^ School of Psychology Liverpool John Moores University Liverpool UK; ^2^ Division of Geriatrics and Gerontology Department of Medicine University of Wisconsin Madison Wisconsin USA; ^3^ Wisconsin Alzheimer's Disease Research Center School of Medicine and Public Health University of Wisconsin–Madison Madison Wisconsin USA; ^4^ Geriatric Research, Education and Clinical Center William S. Middleton Memorial Veterans Hospital Madison Wisconsin USA; ^5^ Department of Medicine University of Wisconsin–Madison Madison Wisconsin USA; ^6^ Wisconsin Alzheimer's Institute School of Medicine and Public Health University of Wisconsin–Madison Madison Wisconsin USA; ^7^ Department of Neurology University of Wisconsin–Madison Madison Wisconsin USA; ^8^ Department of Psychiatry and Neurochemistry Institute of Neuroscience and Physiology the Sahlgrenska Academy at the University of Gothenburg Mölndal Sweden; ^9^ Clinical Neurochemistry Laboratory Sahlgrenska University Hospital Göteborg Sweden; ^10^ Department of Neurodegenerative Disease Institute of Neurology, UCL London UK; ^11^ UK Dementia Research Institute, UCL London UK; ^12^ Hong Kong Center for Neurodegenerative Diseases, Science Park Hong Kong China; ^13^ School of Sports Exercise and Health Sciences Loughborough University Loughborough UK; ^14^ Department of Communication Sciences and Disorders University of Wisconsin–Madison Madison Wisconsin USA

**Keywords:** Alzheimer's disease, apolipoprotein E ε4 allele, biomarker, cerebrospinal fluid, hormone therapy, menopause

## Abstract

**INTRODUCTION:**

Menopausal hormone therapy (MHT), along with the apolipoprotein E (*APOE*) ε4 allele, has been suggested as a possible risk factor for Alzheimer's disease (AD). However, the relationship between MHT and cerebrospinal fluid (CSF) biomarkers is unknown: we investigated this association, and whether *APOE* ε4 carrier status moderates it.

**METHODS:**

In an observational study of 136 cognitively unimpaired female participants (*M*_age_ = 66.0; standard deviation = 6.3), we examined whether MHT use alone or in interaction with *APOE* ε4 carrier status was associated with CSF levels of phosphorylated tau (p‐tau), amyloid beta (Aβ)40, Aβ42, p‐tau/Aβ42, and Aβ42/40 ratios.

**RESULTS:**

Significant interactions were found between *APOE* ε4 and MHT use for CSF biomarkers. *APOE* ε4 carriers who were MHT users showed worse levels of CSF p‐tau/Aβ42 and Aβ42/40 ratios than all other users and non‐users.

**DISCUSSION:**

The presence of both *APOE* ε4 and MHT may be associated with elevated amyloid deposition and AD pathology in this sample of participants who demonstrated high familial AD risk.

**Highlights:**

- Significant interactions were found between apolipoprotein E (*APOE*) ε4 and menopausal hormone therapy (MHT) use for cerebrospinal fluid (CSF) phosphorylated tau (p‐tau)/amyloid beta (Aβ)42 and Aβ42/40 ratios.
- *APOE* ε4 carriers who were MHT users showed worse levels of CSF biomarkers than non‐users and non‐carriers, both users and non‐users.
- Younger age at MHT initiation was associated with worse levels of the p‐tau/Aβ42 and Aβ42/40 ratios in carriers only.
- The presence of both *APOE* ε4 carriage and MHT use may be associated with elevated amyloid deposition and AD pathology.
- Further studies with larger sample sizes are necessary to confirm the differences observed in the current study.

## 1 BACKGROUND

Alzheimer's disease (AD) is the most prevalent form of dementia, with two thirds of those affected being women.^1^, ^2^ Although the underlying mechanisms for the sex differences remain unclear,^3^, ^4^, ^5^ hormonal changes during menopause have been proposed as a contributing factor.^6^ Specifically, estrogen has been found to be neuroprotective, and its loss due to menopause is suggested to play a fundamental role in the higher prevalence of AD in women.^6^, ^7^, ^8^ Consequently, the effects of menopausal hormone therapy (MHT) on cognition and AD risk have been investigated, but results have been contradictory. While observational studies have suggested that MHT might reduce AD risk,^9^, ^10^, ^11^, ^12^ a large randomized controlled trial (RCT), the Women's Health Initiative Memory Study (WHIMS), found greater brain atrophy^12^ and increased dementia risk in women who initiated MHT more than a decade after menopause.^13^, ^14^

Conflicting results might be partially due to differences in the timing of MHT initiation (see Morgan et al.^15^ for details). When MHT is initiated near menopause, some observational studies report an association between MHT use and reduced AD risk,^16^, ^17^ while another observational study reported MHT exposure was associated with an increased rate of dementia diagnosis.^18^ Consistent with this finding, greater increases in ventricular volumes, indicative of brain aging, were observed in MHT users compared to placebo, especially when MHT was started later in life,^19^ though this effect was temporary.^20^ Using positron emission tomography (PET), a cross‐sectional study found that in women with high neocortical amyloid beta (Aβ), MHT use was associated with increased tau PET levels, particularly in those initiating MHT > 5 years post‐menopause.^21^ Conversely, RCTs of MHT initiated close to menopause have shown that MHT did not improve or impair cognitive function.^22^, ^23^, ^24^

In addition to timing, apolipoprotein E (*APOE*) genotype has been suggested as a potential moderator between MHT and AD risk.^6^, ^25^ The *APOE* ε4 allele is considered the most important genetic risk factor for AD,^26^, ^27^ with women appearing more susceptible to its negative effects than men.^7^, ^28^, ^29^ While some RCTs, such as WHIMS, did not include *APOE* in the analyses,^12^, ^13^, ^14^, ^22^ others reported *APOE* did not influence cognitive outcomes.^23^, ^24^ However, one observational study indicated that *APOE* ε4 carriers who used MHT showed improved cognitive performance and larger entorhinal and amygdala volumes,^8^ though another reported that *APOE* ε4 homozygote women using MHT had lower hippocampal, parahippocampal, and thalamus volumes than non‐users.^30^ Recently menopausal *APOE* ε4 carriers have shown increased amyloid burden relative to premenopausal women and men,^31^, ^32^ with a PET study revealing that MHT use in recently menopausal women was associated with reduced Aβ deposition compared to placebo, particularly in *APOE* ε4 carriers.^33^ Similarly, a study of plasma AD biomarkers found that *APOE* ε4 carriers receiving MHT had less reduction in plasma Aβ42/phosphorylated tau (p‐tau)231 ratios than those on placebo, and that within MHT users, carriers showed greater reductions of Aβ42 levels than non‐carriers, indicating less likely progression toward AD pathology after 6 months.^34^

Overall, the effects of MHT on the brain appear to depend on multiple factors, such as timing and *APOE* genotype, yet findings remain inconsistent. Although the relationship between MHT and AD biomarkers has been explored, studies using cerebrospinal fluid (CSF) biomarkers are lacking. Given the strong predictive value of CSF biomarkers, including the p‐tau/Aβ42 and Aβ42/40 ratios, for brain amyloid pathology,^35^, ^36^ examining potential differences between MHT users and non‐users is necessary. Therefore, this study aims to address a gap in the literature by investigating how the interaction between MHT use and *APOE* ε4 carrier status may influence CSF biomarkers of AD. Specifically, we investigated whether MHT alone or in interaction with *APOE* ε4 carrier status are associated with CSF levels of the p‐tau181/Aβ42 and Aβ42/Aβ40 ratios, or with the individual markers from which these ratios are derived. Additionally, a secondary analysis examined if age at MHT initiation is associated with CSF levels of AD biomarkers, and whether *APOE* ε4 carrier status moderates these associations.

## 2 METHODS

### 2.1 Participants

Data were extracted from the Wisconsin Registry for Alzheimer's Prevention (WRAP), an ongoing longitudinal cohort study based at the University of Wisconsin–Madison, USA, in which participants attend regular visits; the first follow‐up occurs 2 to 4 years after baseline and then every 2 years (for details, see Johnson et al.^37^ and Sager et al.^38^). The initial strategy of the WRAP study was to enroll a sample enriched for AD risk by enlisting the adult children of person's diagnosed with dementia at a university‐based clinic.^37^ The resultant convenience sample represented a group at high risk due to parental history, but who also exhibited low level of risk due to the social, lifestyle/behavioral, and environmental exposures.

At each study visit, participants completed self‐report questionnaires on demographics, health history, and lifestyle, in addition to clinical assessments and a neuropsychological test battery (for a full list of procedures and tests, see Johnson et al.^37^). Participants were classified after each study visit as cognitively unimpaired–stable (CUS), cognitively unimpaired–declining (CUD), mild cognitive impairment (MCI), or dementia via a two‐tiered consensus conference diagnosis (as described in Koscik et al.^39^). For the present study, participants self‐identifying as female were selected based on having completed at least two visits, one in which they underwent a lumbar puncture (LP) and one in which they completed specific women's questions (described in section 2.2). In all cases, participants’ sex was presumed to have been assigned female at birth, that is, self‐identified sex and sex at birth were concordant. Within the women's questions, participants who answered “don't know” or “unknown” to the MHT use or age at menopause questions were excluded. Finally, as responses to questions about MHT use were self‐reported, only participants classified as cognitively unimpaired at the time of MHT use data collection were included, to ensure the reliability of their responses.

**RESEARCH IN CONTEXT**

1. **Systematic review**: We searched articles through online databases such as PubMed and Google Scholar. We focused on studies investigating the association between menopausal hormone therapy (MHT) and Alzheimer's disease (AD), along with potential moderating factors.
2. **Interpretation**: We report that in women carrying at least one apolipoprotein E ε4 allele, MHT use may be associated with elevated amyloid deposition and AD pathology, as shown by cerebrospinal fluid (CSF) levels of the phosphorylated tau/amyloid beta (Aβ)42 and Aβ42/40 ratios. This is the first study to investigate the association between MHT use and CSF biomarkers of AD.
3. **Future directions**: Further studies with larger sample sizes are necessary to confirm the differences observed in the current study.

From the total pool of 1750 participants (for a consort flow diagram, see Figure S1 in supporting information), 141 participants fulfilled the above inclusion criteria. However, women with ε2/ε4 genotype (*n* = 5) were excluded, as the ε2 allele is considered protective while the ε4 is a risk allele.^8^ From the remaining 136 participants, four reported their race as Black or African American, one reported their ethnicity as Hispanic, and 131 identified as non‐Hispanic and White. CSF levels of the biomarkers were obtained from the most recent LP visit of each participant, while all available MHT use data, including any reported changes up to the most recent LP visit, were analyzed. All activities for this study were approved by the institutional review board of the University of Wisconsin–Madison and completed in accordance with the Declaration of Helsinki. All participants provided informed consent prior to testing.

### 2.2 Exposure

As part of the assessment, participants completed specific questions for women, answers were given at a baseline visit, and any changes relative to baseline were collected at subsequent visits. Participants were asked one question regarding whether they were using hormone therapy (any form of estrogen with or without progesterone) at that time. The response options were: 1 = yes; 2 = no, but I have in the past; 3 = no, never; and 4 = don't know. Current and past MHT use were pooled into a single category, “MHT users,” for comparison to participants with no use. MHT use (non‐user/user) was included as a predictor in the main analyses, which investigated the associations between MHT use and CSF biomarkers. Of the 86 MHT users included in the analyses, 20 were still using MHT at the time of their most recent LP; 30 had used MHT either before baseline assessment or intermittently during/between WRAP visits; and 36 had used MHT only before baseline assessment, with no further use afterward. Although past use prior to baseline could introduce potential inaccuracies due to recall bias, this risk was mitigated by including only women who were cognitively unimpaired at the time of self‐report. Additionally, further details such as the medication name, form, duration of MHT, and the age at initiation, were requested to improve recall accuracy.

For the secondary analyses, which investigated the associations between age at MHT initiation and CSF biomarkers, specific information regarding MHT use was included. For past users, detailed data were collected regarding the name of the MHT medication, its form, duration of use, and the age at initiation. For current users, the same data were gathered at baseline, and any changes during follow‐up visits were registered. This information was cross‐referenced with self‐reported current medications at all available visits for each participant. MHT medications were categorized into four groups: estrogen, conjugated equine estrogen (cEE), estrogen and progesterone, or cEE and progesterone. For combined medications, they were taken either as a single combined medication or as two medications at the same time. Participants used the following MHT forms: pill, cream, ring, or combined forms. Combined forms could involve the simultaneous or sequential use of different forms. The duration of MHT use was calculated in years, accounting for all self‐reported past and current usage up to the most recent LP visit. While dosage information was collected for current MHT users at the time of each visit, it was not requested for past medications; therefore, dosage was not included in the analyses. Similarly, although information on possible side effects was collected, complete data were not available and, therefore, not analyzed. The age at initiation was self‐reported for each medication. Out of 86 MHT users, 9 participants did not provide age at MHT initiation and thus, were excluded from the secondary analyses. From the remaining 77 MHT users, 3 past users responded they did not remember the medication name, and 1 participant, also a past user, did not provide name, form, or duration; due to the importance of controlling for medication type, they were excluded from the secondary analyses. The rationale for excluding participants with missing data was to avoid potential biases from imputation.

There was one question related to MHT use in the questionnaire that was not included in the analysis: MHT initiation relative to menopause. The response options were 1 = still in menopause, 2 = within 1 year after menopause, 3 = > 1 year after menopause, 4 = > 5 years after menopause, or 5 = don't know. Because these response options were non‐linear, and the number of MHT users was already limited, including this categorical variable as a predictor would have further restricted the group sizes. Therefore, age at MHT initiation was used as a predictor instead.

In addition to MHT use data, other variables of interest for the current study were analyzed. Participants reported their age at their last menstrual period, to which we added 1 year to determine the age at menopause,^40^ and age at menarche. We collected a history of surgeries, including oophorectomy (partial, one, or both ovaries), hysterectomy (partial or entire uterus), or both, along with the age at surgery and whether they ceased having periods post‐surgery.

### 2.3 Genotyping

DNA was extracted from whole blood. Samples were aliquoted on 96‐well plates for the determination of *APOE* genotypes. Women were classified into ε4 carrier and non‐carrier groups based on their *APOE* genotype (referred to as *APOE* ε4): the ε4 carrier group included participants with either ε3/ε4 or ε4/ε4 genotype combinations, and the non‐carrier group included those with either ε2/ε2, ε2/ε3, or ε3/ε3 genotypes.^41^, ^42^ As mentioned, women with ε2/ε4 genotype (*N* = 5) were excluded from the study, as the ε2 allele is considered protective while the ε4 is a risk allele,^8^ and as this was a small number of participants for subgroup analyses.

### 2.4 CSF collection

CSF was extracted using a Sprotte 24‐ or 25‐gauge spinal needle, under fasting conditions. During each LP visit, 22 mL of CSF was extracted, which was then combined, mixed, centrifuged, and aliquoted into tubes of 1.5 mL capacity. These tubes were stored within 30 minutes at −80°C (for more details on the CSF procedure, see Van Hulle et al.^43^).

### 2.5 Biomarker measurements

CSF biomarkers were measured with Roche NeuroToolKit assays (Roche Diagnostics International Ltd.), using the same batch of reagents under strict quality control procedures. Elecsys Aβ42, Aβ40, and p‐tau (181P) were performed on a cobas e 601 analyzer, as previously described.^43^ The primary outcomes of this study were the ratios of p‐tau/Aβ42 and Aβ42/Aβ40, as these measures have been shown to be predictive of brain amyloid pathology, and to be superior to individual markers compared to amyloid PET concordance.^35^, ^36^ Secondary outcomes included the individual CSF biomarkers from which these ratios are derived: p‐tau181, Aβ40, and Aβ42.

### 2.6 Control variables

Demographic variables included were age at most recent LP and years of education as continuous variables, and race/ethnicity, which was entered as a categorical variable with three levels: non‐Hispanic and White, Black/African American, or Hispanic. To account for the elapsed time between menopause age and LP visit, we subtracted age at menopause to age at LP, and entered it in the models as a continuous variable. All women included in this study reported being postmenopausal at the visit closest in time to most recent LP. To account for history of surgeries, which included oophorectomy (partial, one, or both ovaries), hysterectomy (partial or entire uterus), or both, we entered history as a dichotomized variable, by pooling women who had any of the two surgeries as having a history of surgery, versus those who did not. Considering that longer reproductive period has been found to be associated with CSF biomarkers of AD,^44^ we intended to include it as a covariate. However, because reproductive period is calculated by subtracting the age at menarche from the age at menopause, issues with multicollinearity were observed with elapsed time between menopause age and LP, and with age at most recent LP, resulting in problematic variance inflation factors (VIFs) for the three variables (VIF > 5). Thus, age at menarche was included, instead of reproductive period. By doing so, the statistical analyses controlled for age at LP and age at menarche, while also indirectly controlling for age at menopause, by including the elapsed time between age at menopause and LP.

To control for other possible confounders, we also included a multivariable lifestyle‐based dementia risk score, the Lifestyle for Brain Health (LIBRA^45^) index, as a covariate. The index consists of the following risk factors: physical inactivity, smoking, depression, hypertension, obesity, diabetes, hypercholesterolemia, coronary artery disease, and renal disease. Protective factors include low‐to‐moderate alcohol use, high cognitive activity, and healthy diet. In WRAP, longitudinal data on diet was not available and thus, it was not included in the index. A sum score was calculated for each participant based on the weighted factors, and LIBRA risk groups were determined using baseline LIBRA tertiles: low risk, moderate risk, and high risk (for details on the operationalization of the LIBRA index in the WRAP dataset, see Cody et al.^46^); LIBRA risk group was entered in the model as a categorical variable. There were two main reasons for including the LIBRA index. First, several studies have shown that LIBRA is predictive of cognitive decline and risk of dementia,^45^, ^47^, ^48^ even in *APOE* ε4 carriers and non‐carriers.^49^ Second, by including this composite measure, in contrast to entering each of risk and protective factor separately, we tried to avoid overfitting the models, especially as the sample sizes were relatively small. With this approach, we intended to control for as many risk factors for dementia as possible, both non‐modifiable factors (e.g., age and *APOE* ε4 carrier status) and modifiable risk and protective factors, as assessed with the LIBRA index.

For the main analyses, in which both MHT users and non‐users were included, covariates were age at most recent LP, elapsed time between menopause and LP, years of education, race/ethnicity (non‐Hispanic White as reference category), history of surgery (no as reference) at LP, age at menarche, and LIBRA risk group at LP (low as reference). For the secondary analyses, in which only MHT users were included, covariates were the same as for the main analyses, along with specific MHT‐related variables: MHT medication (estrogen as reference), MHT form (pill as reference), and MHT duration in years (for details of how these were operationalized, see section 2.2). All the continuous covariates were centered by subtracting the mean of each variable from its observed values.

### 2.7 Statistical analysis

Assumptions of normality and homoscedasticity were checked, along with Q‐Q plots; CSF levels of the p‐tau/Aβ42 ratio and Aβ42 were log10 transformed due to non‐normal distribution. We ran Student tests, Mann–Whitney tests, Fisher exact tests, or Pearson chi‐square tests where appropriate, to determine whether there were differences between MHT users and non‐users in the control variables.

Linear models (LMs) were used to explore the association between MHT use and levels of CSF biomarkers. The primary outcomes were the p‐tau/Aβ42 and Aβ42/40 ratios, and secondary outcomes included individual biomarkers, Aβ42, Aβ40, and p‐tau. First, separate LMs were fitted for each CSF biomarker as outcome; MHT use (non‐users as reference) served as the predictor; and the covariates, which included *APOE* ε4 carrier status (non‐carriers as reference), were also entered. Second, to explore whether *APOE* ε4 carrier status influences the associations between MHT use and CSF biomarker levels, we included an interaction term between MHT use and *APOE* ε4 carrier status to the previous model. We report unstandardized coefficients (B), standard errors (SEs), *P* values (alpha set to 0.05) and confidence intervals (CIs). Additionally, partial Cohen *ƒ* ^2^ was used to assess the unique contribution of each predictor and the interaction term to the explained variance in CSF biomarkers, with thresholds for small (0.02), medium (0.15), and large (0.35) effects.^50^ Only participants with complete CSF biomarker levels, MHT use data (users or non‐users), and genotyping were included in the analyses; there were no missing values for the covariates.

If significant interactions were found, post hoc analyses were conducted to explore differences in estimated marginal means between specific subgroups defined by combinations of MHT use and *APOE* ε4 status. The subgroups included *APOE* ε4 non‐carriers who did not use MHT (ε4–MHT–), *APOE* ε4 non‐carriers who used MHT (ε4–MHT+), *APOE* ε4 carriers who did not use MHT (ε4+MHT–), and *APOE* ε4 carriers who used MHT (ε4+MHT+). All possible subgroup comparisons were made to provide a detailed understanding of the potential differences between groups, and the Benjamini–Hochberg (BH) procedure was applied to control the false discovery rate (FDR) for multiple comparisons.^51^ Cohen *d* was used as a measure of effect size, with thresholds for small (0.20), medium (0.50), and large (0.80) effects.^52^

As a secondary analysis, we investigated the association between age at MHT initiation and CSF biomarker levels in MHT users, using linear regression. The first model included the primary outcomes, p‐tau/Aβ42 and Aβ42/40 ratios separately, with age at MHT initiation as the predictor. The covariates included those from the main analyses, in addition to MHT medication, MHT form, and MHT duration. To explore whether *APOE* ε4 carrier status influences the association between age at MHT initiation and CSF biomarker levels, an interaction term between age at MHT initiation and *APOE* ε4 carrier status was added to the previous model. We report regression coefficients, standard errors, *P* values (alpha set to 0.05), CIs, and partial Cohen *ƒ* ^2^. If the interaction was found to be significant, a simple slope analysis was then conducted to determine the slopes for age at MHT initiation by *APOE* ε4 carrier status.

For all the models tested, influential data points on model outputs were inspected using Cook's distance; no data points had a Cook's distance ≥ 1. In addition, all the models were checked for multicollinearity and none of the variables had a VIF > 2. Statistical analyses were carried out using R software, version 4.3.2. Mann–Whitney tests, Student *t* tests, Fisher exact tests, Pearson chi‐square tests, and linear model analyses were performed with the R Stats Package. VIFs were calculated with the “car” package. Post hoc and simple slope analyses were conducted using the “emmeans” package; effect sizes were calculated using “effectsize.” Figures were plotted with the “interactions” package. All the packages are available at http://cran.r‐project.org/web/packages.

## 3 RESULTS

### 3.1 Comparison of the control variables

Table 1 reports means and standard deviations, or count and percentages, of the study characteristics, described by whole sample and MHT use at LP visit. There were no significant differences in education years, age at menarche, or the percentages of race, or of those with history of surgery, or of *APOE* ε4 carriers and non‐carriers, or of parental history of AD, between MHT users and non‐users. However, MHT users were significantly older at most recent LP (*P <* 0.001), were younger at menopause (*P *= 0.043), and had longer elapsed times between menopause and most recent LP (*P *< 0.001), than non‐users. There were also significant differences between groups in LIBRA risk group percentages (*P* = 0.014), while the percentage of non‐users at high risk was higher than that of users, the opposite was observed for those at moderate risk.

**TABLE 1:** Means (standard deviations) or count (percentage) of covariates by whole sample and MHT usage.

	Total (*n* = 136)	MHT use		
		Users (*n* = 86)	Non‐users (*n* = 50)	value
Age at LP	65.95 (6.3)	67.59 (6.2)	63.13 (5.5)	< 0.001
Education years	15.97 (2.0)	16.07 (2.0)	15.80 (2.0)	0.520
Age at menopause	50.17 (5.8)	49.52 (6.2)	51.28 (4.8)	0.043
Elapsed time	15.78 (8.2)	18.07 (8.2)	11.85 (6.5)	< 0.001
Age at menarche	36.79 (6.1)	12.36 (1.3)	12.42 (1.7)	0.806
Surgery history (yes)	52 (38.2%)	34 (39.5%)	18 (36%)	0.683
Race				0.356
Non‐Hispanic White	131 (96.3%)	84 (97.7%)	47 (94%)	
Black/African American	4 (2.9%)	2 (2.3%)	2 (4%)	
Hispanic	1 (0.7%)	0	1 (2%)	
LIBRA index				0.014
Low risk	55 (40.4%)	35 (40.7%)	20 (40%)	
Moderate risk	44 (32.4%)	34 (39.5%)	10 (20%)	
High risk	37 (27.2%)	17 (19.8%)	20 (40%)	
*APOE* ε4 carrier status				0.810
Carrier	48 (35.3%)	31 (36%)	17 (34%)	
Non‐carrier	88 (64.7%)	55 (64%)	33 (66%)	
Parental history of AD (yes)	102 (75%)	64 (74.4%)	38 (76%)	0.837

*Note*: Parental history of AD and age at menopause are reported for reference. Statistical tests were conducted to check for differences between MHT users and non‐users, *P* values are reported. Elapsed time: time elapsed between age at menopause and lumbar puncture, in years.

Abbreviations: AD, Alzheimer's disease; *APOE*, apolipoprotein E; LIBRA, the Lifestyle for Brain Health index; LP, lumbar puncture; MHT, menopausal hormone therapy.

### 3.2 The association between MHT use and CSF biomarker ratios is moderated by *APOE* ε4 carrier status

For the primary outcomes, the main effects models revealed no significant associations between MHT use and CSF (log‐transformed) p‐tau/Aβ42 ratio (*P* = 0.747) or CSF Aβ42/40 ratio (*P* = 0.796) levels; for both outcomes, significant covariates were age at LP, race (non‐Hispanic White vs. Black/African American) and *APOE* ε4 carrier status (ε4− vs. ε4+). When the interaction term between MHT use and *APOE* ε4 carrier status was entered into the models, the analyses showed the interaction was significant for the CSF (log‐transformed) p‐tau/Aβ42 ratio (*B* = 0.207, SE = 0.085, *P* = 0.016, 95% CI: [0.039, 0.374], *ƒ* ^2^ = 0.05) and the Aβ42/Aβ40 ratio (*B* = −0.017, SE = 0.006, *P* = 0.008, 95% CI: [−0.030, −0.005], *ƒ* ^2^ = 0.06). In the interaction models, significant covariates were age at LP, race (non‐Hispanic White vs. Black/African American) and LIBRA (low vs. moderate risk). We report the full main effects and interaction models for each outcome in Table 2.

**TABLE 2:** Main effects and interaction models with (log‐transformed) CSF p‐tau/Aβ42 and Aβ42/40 ratios as outcomes.

	CSF p‐tau/Aβ42 ratio		CSF Aβ42/40 ratio	
	Main effects	Interaction	Main effects	Interaction
	*B* (SE)	*B* (SE)	*B* (SE)	*B* (SE)
Constant	−1.693^***^ (0.048)	−1.655^***^ (0.050)	0.064^***^ (0.004)	0.061^***^ (0.004)
Age at LP	0.014^**^ (0.005)	0.015^**^ (0.005)	−0.001^**^ (0.000)	−0.001^**^ (0.000)
Education years	0.014 (0.010)	0.015 (0.010)	−0.001 (0.001)	−0.001 (0.001)
Elapsed time	−0.004 (0.004)	−0.005 (0.004)	0.000 (0.000)	0.000 (0.0003)
Race (Black)	0.270^*^ (0.119)	0.299^*^ (0.117)	−0.018^*^ (0.009)	−0.020^*^ (0.009)
Race (other)	−0.107 (0.230)	−0.149 (0.226)	0.007 (0.017)	0.010 (0.017)
Surgery (yes)	0.023 (0.043)	0.041 (0.043)	−0.004 (0.003)	−0.005 (0.003)
Age at menarche	0.017 (0.014)	0.017 (0.014)	−0.001 (0.001)	−0.001 (0.001)
LIBRA (moderate)	−0.075 (0.047)	−0.084 (0.046)	0.006 (0.003)	0.007^*^ (0.003)
LIBRA (high)	−0.087 (0.052)	−0.083 (0.051)	0.004 (0.004)	0.004 (0.004)
*APOE* ε4 (+)	0.174^***^ (0.041)	0.041 (0.068)	−0.012^***^ (0.003)	−0.001 (0.005)
MHT(+)	0.014 (0.044)	−0.054 (0.052)	−0.001 (0.003)	0.005 (0.004)
*APOE* ε4 (+):MHT(+)		0.207^*^ (0.085)		−0.017^**^ (0.006)
*R* ^2^ (adjusted)	0.238 (0.171)	0.274 (0.203)	0.218 (0.148)	0.262 (0.189)
*F* statistic	3.527^***^ df = 11, 124	3.861^***^ df = 12, 123	3.139^***^ df = 11, 124	3.630^***^ df = 12, 123

Abbreviations: Aβ, amyloid beta; *APOE*, apolipoprotein E; CSF, cerebrospinal fluid; df, degrees of freedom; LIBRA, the Lifestyle for Brain Health index; LP, lumbar puncture; MHT(+), menopausal hormone therapy (+ represents users); p‐tau, phosphorylated tau; SE, standard error.

* *P* < 0.05.

** *P* < 0.01.

*** *P* < 0.001.

**FIGURE 1:**
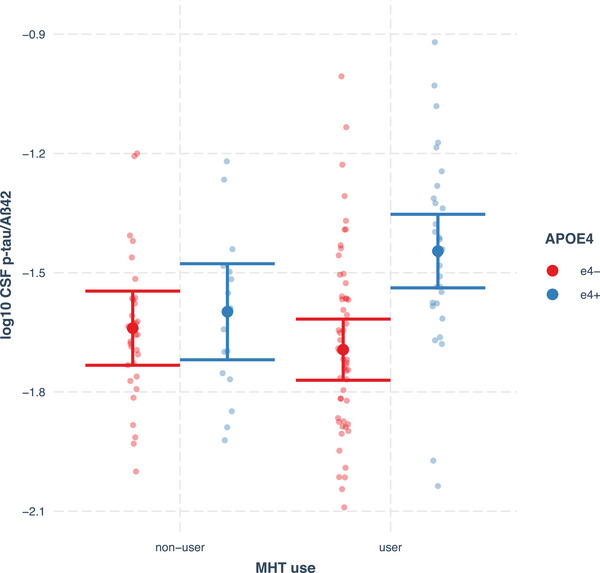
Estimated marginal means of CSF levels of the p‐tau/Aβ42 ratio (log10 transformed) by MHT use and *APOE* ε4 carrier status, controlling for the covariates, with 95% confidence interval. Estimated marginal means and CIs of CSF p‐tau/Aβ42 ratio in each group (back‐transformed): ε4−MHT− = 0.023, 95% CI: [0.015, 0.034]; ε4+MHT− = 0.025, 95% CI: [0.016, 0.040]; ε4−MHT+ = 0.020, 95% CI: [0.014, 0.030]; ε4+MHT+ = 0.036, 95% CI: [0.023, 0.055]. Post hoc pairwise group comparisons: ε4+MHT+ versus ε4−MHT+, adjusted *P* < 0.001; ε4+MHT+ versus ε4−MHT−, adjusted *P* = 0.003; ε4+MHT+ vs. ε4+MHT−, adjusted *P* = 0.069; ε4−MHT− versus ε4+MHT−, adjusted *P* = 0.541; ε4−MHT− versus ε4−MHT+, adjusted *P* = 0.357; ε4+MHT− versus ε4−MHT+, adjusted *P* = 0.226. Aβ, amyloid beta; *APOE*, apolipoprotein E; CI, confidence interval; CSF, cerebrospinal fluid; ε4−, non‐carriers; ε4+, carriers; MHT, menopausal hormone therapy; p‐tau, phosphorylated tau.

**FIGURE 2:**
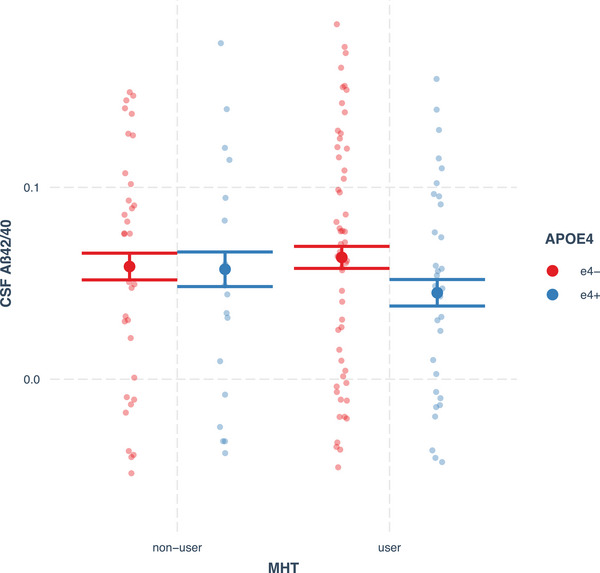
Estimated marginal means of CSF levels of the Aβ42/40 ratio (pg/mL) by MHT use and *APOE* ε4 carrier status, controlling for the covariates, with 95% confidence interval. Estimated marginal means and CIs of CSF Aβ42/40 ratio in each group: ε4−MHT− = 0.058, 95% CI: [0.045, 0.071]; ε4+MHT− = 0.057, 95% CI: [0.042, 0.072]; ε4−MHT+ = 0.063, 95% CI: [0.050, 0.076]; ε4+MHT+ = 0.045, 95% CI: [0.031, 0.059]. Post hoc pairwise group comparisons: ε4+MHT+ versus ε4−MHT+, adjusted *P* < 0.001; ε4+MHT+ versus ε4−MHT−, adjusted *P* = 0.006; ε4+MHT+ versus ε4+MHT−, adjusted *P* = 0.046; ε4−MHT− versus ε4+MHT−, adjusted *P* = 0.777; ε4−MHT− versus ε4−MHT+, adjusted *P* = 0.262; ε4+MHT− versus ε4−MHT+, adjusted *P* = 0.262. Aβ, amyloid beta; *APOE*, apolipoprotein E; CI, confidence interval; CSF, cerebrospinal fluid; ε4−, non‐carriers; ε4+, carriers; MHT, menopausal hormone therapy

The estimated marginal means of each group revealed that the ε4+MHT+ group had the worst CSF levels of p‐tau/Aβ42 and Aβ42/40 ratios among all groups (reported in Figures 1 and 2, respectively). Post hoc pairwise comparisons indicated that ε4+MHT+ had significantly higher (worse) CSF levels of the p‐tau/Aβ42 ratio than ε4−MHT+ (adjusted *P* < 0.001, *d* = 1.13), ε4−MHT− (adjusted *P* = 0.003, *d* = 0.88), and ε4+MHT−, yet for this comparison, the difference was no longer significant after applying FDR (unadjusted *P *= 0.034; adjusted *P* = 0.069, *d* = 0.70); no other significant differences between groups were found, see Figure 1. With CSF Aβ42/Aβ40 ratio levels as outcome, pairwise comparisons showed that ε4+MHT+ had significantly lower (worse) levels than ε4−MHT+ (adjusted *P* < 0.001, *d* = −1.13), ε4−MHT− (adjusted *P* = 0.006, *d* = −0.84) and ε4+MHT− (adjusted *P* = 0.046, *d* = −0.75); no other significant differences between groups were found, see Figure 2 for details.

For the secondary outcomes, the main effects models revealed no significant associations between MHT use and CSF (log‐transformed) Aβ42 (*P* = 0.867), Aβ40 (*P* = 0.644), or p‐tau levels (*P* = 0.371); among the covariates, age at LP was significant for p‐tau (*P* < 0.05), *APOE* ε4 was significant for Aβ42 and p‐tau (*P* < 0.05), while LIBRA risk group was significant for Aβ42 and Aβ40 (low vs. high risk, *P* < 0.01). When the interaction term between MHT use and *APOE* ε4 carrier status was entered into the models, the analyses showed the interaction was not significant for CSF Aβ40 (*P* = 0.862), or p‐tau (*P* = 0.348), yet a trend was found for Aβ42 (*B* = −0.149, SE = 0.076, *P* = 0.055, 95% CI: [−0.301, 0.003], *ƒ* ^2^ = 0.03). We report the main effects and interaction models for each outcome in Tables S1, S2, and S3 in supporting information. Post hoc pairwise comparisons indicated that ε4+MHT+ had significantly lower CSF Aβ42 levels (adjusted *P* = 0.007, *d* = 0.76) and higher CSF p‐tau levels than ε4−MHT+ (unadjusted *P* = 0.014, adjusted *P* = 0.056, *d* = 0.57), but no other significant differences were found between groups. Estimated marginal means and pairwise comparisons are reported in Figures S2, S3, and S4 in supporting information.

### 3.3 Associations between age at MHT initiation and CSF biomarkers

Within users with complete MHT use‐related data (*n* = 73; see section 2.2 for details), mean age at MHT initiation was 49.80 (standard deviation [SD] = 5.8; range 30–61), and mean MHT duration was 6.64 (SD = 5.6, range 0–26). From them, 9 used estrogen (12.3%), 23 cEE (31.5%), 26 cEE and progesterone (35.6%), and 15 estrogen and progesterone (20.5%). As for MHT forms, 54 took pills (74%), 6 used cream (8.2%), 3 used ring (4.1%), while 10 used combined forms (13.7%).

The main effects models revealed no significant associations between age at MHT initiation and CSF (log‐transformed) p‐tau/Aβ42 ratio (*P* = 0.384) or Aβ42/40 ratio (*P* = 0.104) levels; for both outcomes, significant covariates were age at LP and *APOE* ε4 carrier status (ε4− vs. ε4+), while race (White vs. Black) was also significant for p‐tau/Aβ42 ratio only, all *P *< 0.05. When the interaction term between age at MHT initiation and *APOE* ε4 carrier status was entered into the models, the analyses showed the interaction was significant for the CSF (log‐transformed) p‐tau/Aβ42 ratio (*B* = −0.030, SE = 0.010, 95% CI: [−0.052, −0.009], *P* = 0.007, *ƒ* ^2^ = 0.15) and the Aβ42/Aβ40 ratio (*B* = 0.002, SE = 0.000, 95% CI: [0.000, 0.004], *P* = 0.013, *ƒ* ^2^ = 0.12). In these models, significant covariates for the p‐tau/Aβ42 ratio were age at LP, education years, age at menarche, and race, while for the Aβ42/40 ratio, age at LP, race, and MHT duration were significant, all *P *< 0.05. We report the full main effects and interaction models for each outcome in Tables S4 and S5 in supporting information.

**FIGURE 3:**
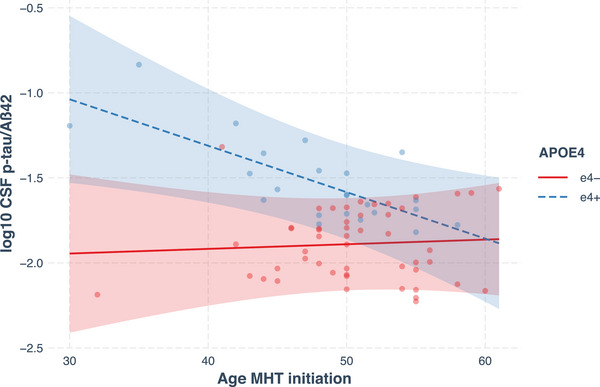
Scatterplot of CSF levels of the p‐tau/Aβ42 ratio (log10 transformed, *y* axis) versus age at MHT initiation (*x* axis), by *APOE* ε4 carrier status. Regression lines and 95% confidence intervals by *APOE* ε4 carrier status, with age at MHT initiation as predictor, and controlling for the covariates. Aβ, amyloid beta; *APOE*, apolipoprotein E; CSF, cerebrospinal fluid; ε4−, non‐carriers; ε4+, carriers; MHT, menopausal hormone therapy; p‐tau, phosphorylated tau

**FIGURE 4:**
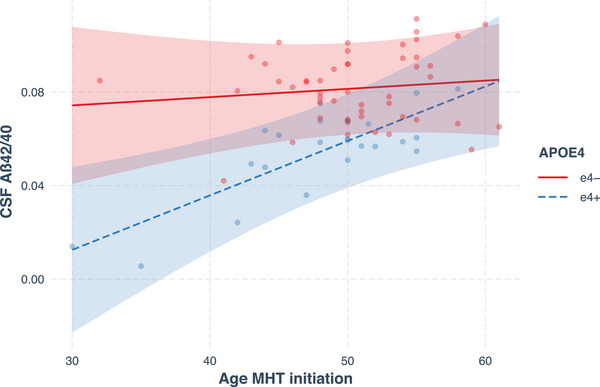
Scatterplot of CSF levels of the Aβ42/40 ratio (untransformed, *y* axis) versus age at MHT initiation (*x* axis), by *APOE* ε4 carrier status. Regression lines and 95% confidence intervals by *APOE* ε4 carrier status, with age at MHT initiation as predictor, and controlling for the covariates. Aβ, amyloid beta; *APOE*, apolipoprotein E; CSF, cerebrospinal fluid; ε4−, non‐carriers; ε4+, carriers; MHT, menopausal hormone therapy.∖

The models with the interaction were analyzed to determine the slopes for age at MHT initiation, calculated separately for ε4− and ε4+ individuals. For ε4+, age at MHT initiation was negatively associated with the log‐transformed CSF p‐tau/Aβ42 ratio levels (*B* = –0.027, SE = 0.011, *P* = 0.015, 95% CI: [–0.049, –0.006]) and positively associated with CSF Aβ42/40 ratio levels (*B* = 0.002, SE = 0.001, *P* = 0.004, 95% CI: [0.001, 0.004]). For ε4–, age at MHT initiation was positively associated with the log‐transformed p‐tau/Aβ42 ratio levels (*B* = 0.003, SE = 0.009, *P* = 0.767, 95% CI: [−0.016, 0.021]) and with Aβ42/40 ratio levels (*B* = 0.000, SE = 0.001, *P* = 0.595, 95% CI: [−0.001, 0.002]), yet these associations were not significant. See Figures 3 and 4 for scatterplots of CSF levels of the p‐tau/Aβ42 ratio and Aβ42/40 ratio versus age at MHT initiation, with regression lines by *APOE* ε4 carrier status.

## 4 DISCUSSION

To our knowledge, this is the first study to examine the association between CSF biomarkers of AD and MHT use. Specifically, we investigated whether MHT alone or in interaction with *APOE* ε4 carrier status were associated with CSF levels of the p‐tau181/Aβ42 and Aβ42/Aβ40 ratios, or with the individual markers from which these ratios are derived. Furthermore, as a secondary analysis, this study also examined if age at MHT initiation was associated with CSF levels of AD biomarkers, and whether *APOE* ε4 carrier status moderated these associations.

Linear regression analyses showed that MHT use was not significantly associated with CSF biomarker levels. However, the interaction between *APOE* ε4 carrier status and MHT use was significant for CSF levels of p‐tau/Aβ42 and Aβ42/40 ratios, being positively associated with the former and negatively associated with the latter. Further analyses revealed that *APOE* ε4 carriers who were or had been MHT users, had significantly higher CSF p‐tau/Aβ42 ratio and lower Aβ42/40 ratio levels than any other group, showing large or medium‐to‐large effect sizes. For both CSF biomarker ratios, the largest effect sizes were observed within MHT users, comparing carriers and non‐carriers, while the same comparison in non‐users yielded no significant differences between them. For the secondary outcomes, that is, CSF p‐tau, Aβ42 and Aβ40 levels, no significant associations were found with MHT use; only for CSF Aβ42 a trend was observed with the interaction. Analyses indicated that *APOE* ε4 carriers who were also MHT users had worse Aβ42 levels than MHT users who were non‐carriers, showing a medium effect size. Even though this difference was limited to MHT users, in contrast to the more extensive differences observed in the ratios, both CSF p‐tau/Aβ42 and Aβ42/Aβ40 have been shown to be predictive of brain amyloid pathology, and to be superior to individual markers compared to amyloid PET concordance.^35^, ^36^

The differences in the associations between MHT use and CSF biomarkers depending on *APOE* ε4 carrier status observed here may be partially explained by the healthy cell bias theory. This theory proposes that while exposure to estrogen might be beneficial for healthy neurons, estrogen might worsen the damage in neurons experiencing pathological changes.^53^ It is possible that in women with at least one ε4 allele, who were already more at risk for AD,^54^ the use of MHT partially contributed to worse levels of CSF biomarkers. This is consistent with a recent study reporting that in *APOE* ε4 homozygote women, MHT use was associated with lower hippocampal, parahippocampal, and thalamus volumes compared to non‐users, either with two or no ε4 alleles.^30^ The authors suggested that these findings might not have been entirely due to *APOE* carrier status, but MHT usage might have also contributed to the reported associations.^30^ In the current study, significant differences within *APOE* ε4 carriers, between MHT users and non‐users, were also found for both ratios, with MHT users showing worse levels than non‐users. Although the difference in CSF Aβ42/40 ratio levels was still significant after correcting for multiple comparisons, this was no longer significant in CSF p‐tau/Aβ42 ratio levels. It should be noted that by comparing subgroups derived from the interaction, the number of women in each subgroup was small, especially of those who were both *APOE* ε4 carriers and non‐users, and thus, these findings should be interpreted with caution.

Our findings differ from other studies investigating the link between MHT use and biomarkers of AD, showing MHT to be beneficial in *APOE* ε4 carriers.^33^, ^34^ However, it is important to note methodological differences between these studies and our own. First, our study was the first to examine MHT use in conjunction with CSF biomarkers, whereas previous studies used other biomarkers of AD (PET or plasma biomarkers). Other notable differences are that in Kantarci et al.,^33^ who reported that MHT use was associated with reduced Aβ deposition and especially in *APOE* ε4 carriers, participants were randomized to MHT, were younger, and there was a lower proportion of carriers. Moreover, no ε4/ε4 carriers were included. Therefore, our participants were arguably at a higher risk of AD pathology than those in Kantarci et al.^33^ In addition, here, women chose whether to use MHT, and the advice they might have received likely varied based on when they went through menopause, particularly before or after the WHIMS study (e.g., Shumaker et al.^13^, ^14^), when potential adverse effects of MHT became widely known.

Depypere et al.^34^ reported that, within *APOE* ε4 carriers, MHT users showed less reduction in plasma Aβ42/p‐tau231 ratio levels compared to *APOE* ε4 carrier non‐users, and that within MHT users, carriers showed greater reductions of Aβ42 levels than non‐carriers, after 6 months. There are several methodological differences that might contribute to the contradicting results. For instance, the prospective longitudinal design of Depypere et al.’s study, alongside their exclusion criteria, which included only women without cardiovascular disease, hypertension, or diabetes, contrasts with our approach of controlling for such factors using a composite index, rather than excluding them. Additionally, the variety of MHT formulations used in our study was broader (see section 2.2 for details), and the duration of MHT use was longer, averaging 6.6 years compared to the 6‐month exposure in theirs.^34^

To date, there is only one study that has explored the association between endogenous estrogen and CSF biomarkers in humans. This study reported that longer exposure to endogenous estrogens, as measured by longer reproductive period, was associated with increased levels of CSF AD biomarkers, specifically, lower levels of Aβ42, lower ratio of Aβ42/Aβ40, and higher levels of p‐tau.^44^ Although this study did not investigate exogenous exposure, as in MHT use, and did not account for *APOE* genotype, current findings are partially consistent with theirs, yet only in MHT users who were *APOE* ε4 carriers.

The present study also investigated if age at MHT initiation is associated with CSF levels of AD biomarkers, and whether *APOE* ε4 carrier status influences it or not. Analyses indicated that the interaction between age at MHT initiation and *APOE* ε4 carrier status was significantly associated with CSF biomarkers. Specifically, younger age at MHT initiation was significantly associated with higher levels of the p‐tau/Aβ42 ratio and lower levels of the Aβ42/40 ratio, in *APOE* ε4 carriers only, while no significant associations were found in non‐carriers. Current findings contrast with two studies reporting that in *APOE* ε4 carriers only, earlier MHT initiation is associated with better outcomes, specifically, less brain aging^41^ and larger hippocampal volumes.^8^ However, none of the two controlled for MHT formulation or delivery form, while in the latter, age at menopause was also not accounted for. Even though we did not find either MHT formulation or form to be associated with CSF biomarkers in the current analyses, certain formulations and delivery forms have been reported to be associated with increased risk of AD.^55^ Further studies with larger sample sizes are required to clarify how timing of MHT initiation is associated with CSF biomarkers of AD, especially in *APOE* ε4 carriers and non‐carriers.

This study's main strength is the availability of CSF biomarkers of AD, along with genotype data, and extensive self‐reported MHT use, menopause, and other confounding factors. However, this study also had limitations that should be noted. One is the age difference between groups, as MHT users were significantly older than non‐users at LP. To account for this difference, we included age as a covariate in all the statistical analyses, and each subgroup's estimated marginal means controlled for age and other covariates. Another limitation is that although self‐reported dosage data were available, this was not available for past users, and thus, it was not included in the analyses. The sample size is another caveat, as it mostly comprised individuals that identified as non‐Hispanic and White, and as in other studies, it was also restricted by the low number of participants who carried at least one ε4 allele, representing 35% of the sample. The effect *APOE* genotype has on AD risk has been reported to vary with ancestry,^56^ which reinforces the importance of investigating genetic risk across a wider spectrum of races/ethnicities. Additionally, due to the nature of the WRAP study (described in section 2.1), the sample comprised a high percentage of participants with parental history of dementia due to AD, yet it did not significantly differ between users and non‐users, and thus, this covariate was not included in the analyses.

This novel study showed that the interaction between *APOE* ε4 carrier status and MHT use was significant for CSF levels of p‐tau/Aβ42 and Aβ42/40 ratios. Specifically, MHT users who were *APOE* ε4 carriers had significantly higher CSF levels of the p‐tau/Aβ42 ratio and lower levels of the Aβ42/40 ratio than users who were non‐carriers and non‐users, regardless of their carrier status. In a secondary analysis, we showed that younger age at MHT initiation was associated with worse CSF p‐tau/Aβ42 and Aβ42/40 ratio levels in *APOE* ε4 carriers only. Current results suggest that in women carrying at least one *APOE* ε4 allele, MHT use may be associated with elevated amyloid deposition and AD pathology.

## CONFLICT OF INTEREST STATEMENT

H.Z. has served on scientific advisory boards and/or as a consultant for Abbvie, Acumen, Alector, Alzinova, ALZPath, Annexon, Apellis, Artery Therapeutics, AZTherapies, CogRx, Denali, Eisai, Nervgen, Novo Nordisk, Optoceutics, Passage Bio, Pinteon Therapeutics, Prothena, Red Abbey Labs, reMYND, Roche, Samumed, Siemens Healthineers, Triplet Therapeutics, and Wave; has given lectures in symposia sponsored by Cellectricon, Fujirebio, Alzecure, Biogen, and Roche; and is a co‐founder of Brain Biomarker Solutions in Gothenburg AB (BBS), which is a part of the GU Ventures Incubator Program (outside submitted work). No other author reports any conflicts of interest or disclosures. Author disclosures are available in the supporting information.

## CONSENT STATEMENT

All participants provided informed consent prior to testing.

## Supporting information



Supporting Information

Supporting Information
