# Supplementary material for: Menopausal hormone therapy is associated with worse levels of Alzheimer's disease biomarkers in APOE ε4‐carrying women: An observational study
Source: Alzheimers Dement. 2025 Jan 9;21(2):e14456. doi: 10.1002/alz.14456 (PMC11848176; doi:10.1002/alz.14456)
Supplement: Supplementary file 1 — Supporting Information [file ALZ-21-e14456-s002.docx]

**Supplementary Material**

**Figure S1.** Consort flow diagram.


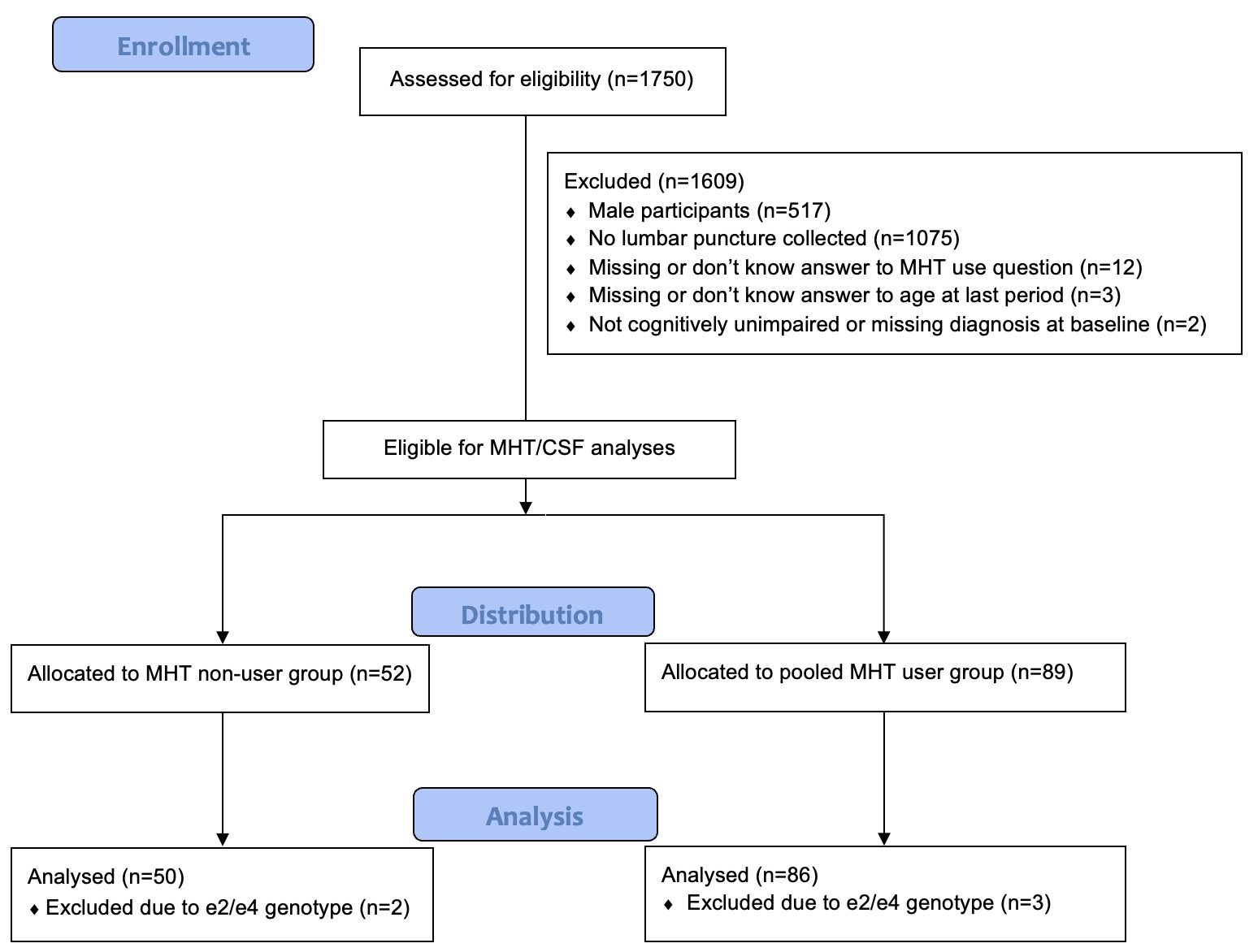


**Figure S2.** Estimated marginal means of CSF levels of Aβ42 (log10 transformed) by MHT use and *APOE*4 carrier status, controlling for the covariates, with 95% confidence interval.

**
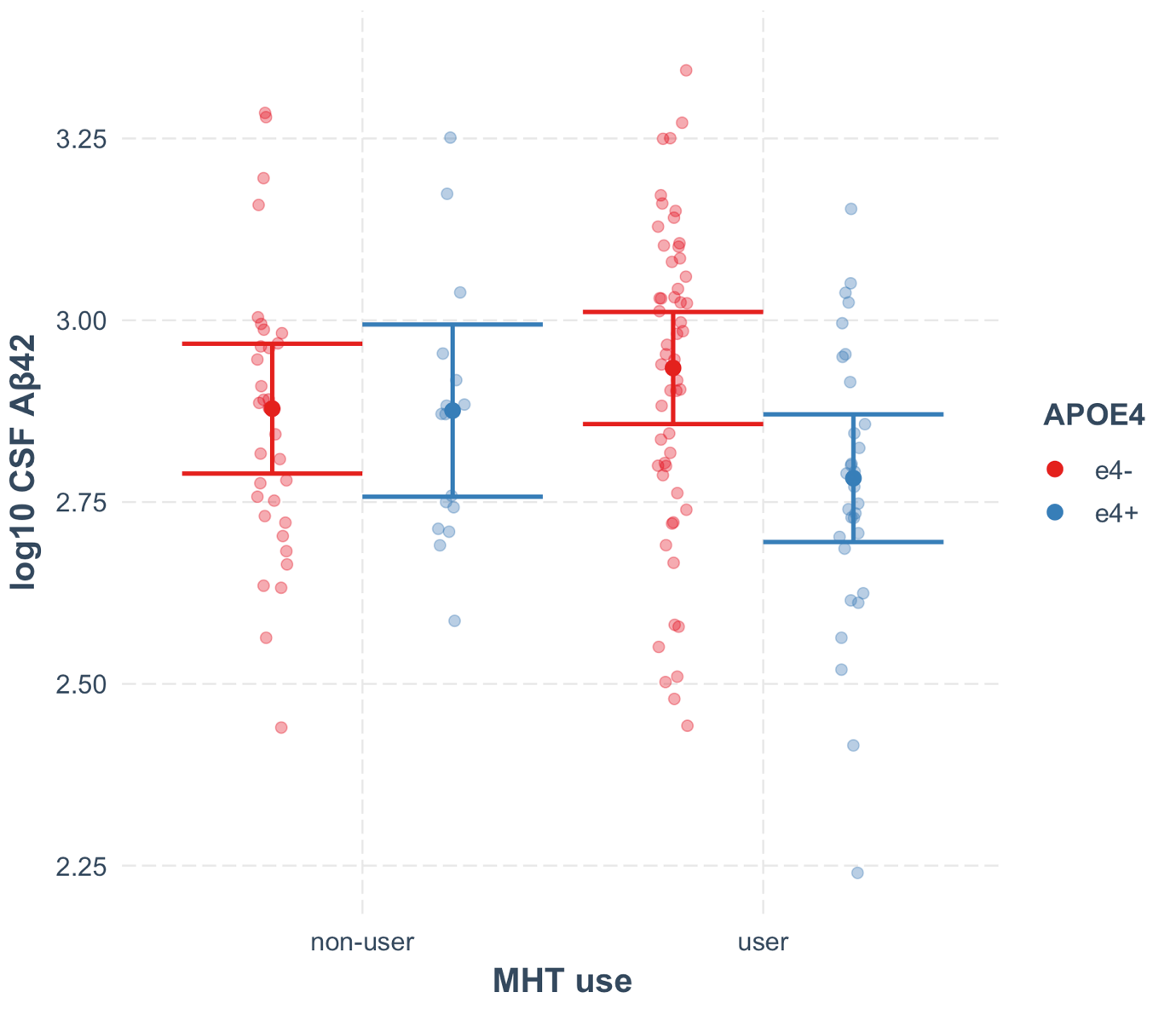
**

*Note:* Estimated marginal means and CIs of CSF Aβ42 levels in each group (back-transformed): e4-MHT- = 664, CI [461, 955]; e4+MHT- = 660, CI [436, 998]; e4-MHT+ = 755, CI [522, 1091]; e4+MHT+ = 533, CI [359, 790]. Abbreviations: Aβ: amyloid-β; APOE4: apolipoprotein E ε4 allele, e4- are non-carriers, e4+ represent carriers; MHT: menopausal hormone therapy. *Post hoc* pairwise group comparisons: ε4+MHT+ vs. ε4-MHT+, adjusted-*p* = .007; ε4+MHT+ vs. ε4-MHT-, adjusted-*p* = .211; ε4+MHT+ vs. ε4+MHT-, adjusted-*p* = .308; ε4-MHT- vs. ε4+MHT-, adjusted-*p* = .965; ε4-MHT- vs. ε4-MHT+, adjusted-*p* = .354; ε4+MHT- vs. ε4-MHT+, adjusted-*p* = .396).

**Figure S3.** Estimated marginal means of CSF levels of Aβ40 by MHT use and *APOE*4 carrier status, controlling for the covariates, with 95% confidence interval.

**
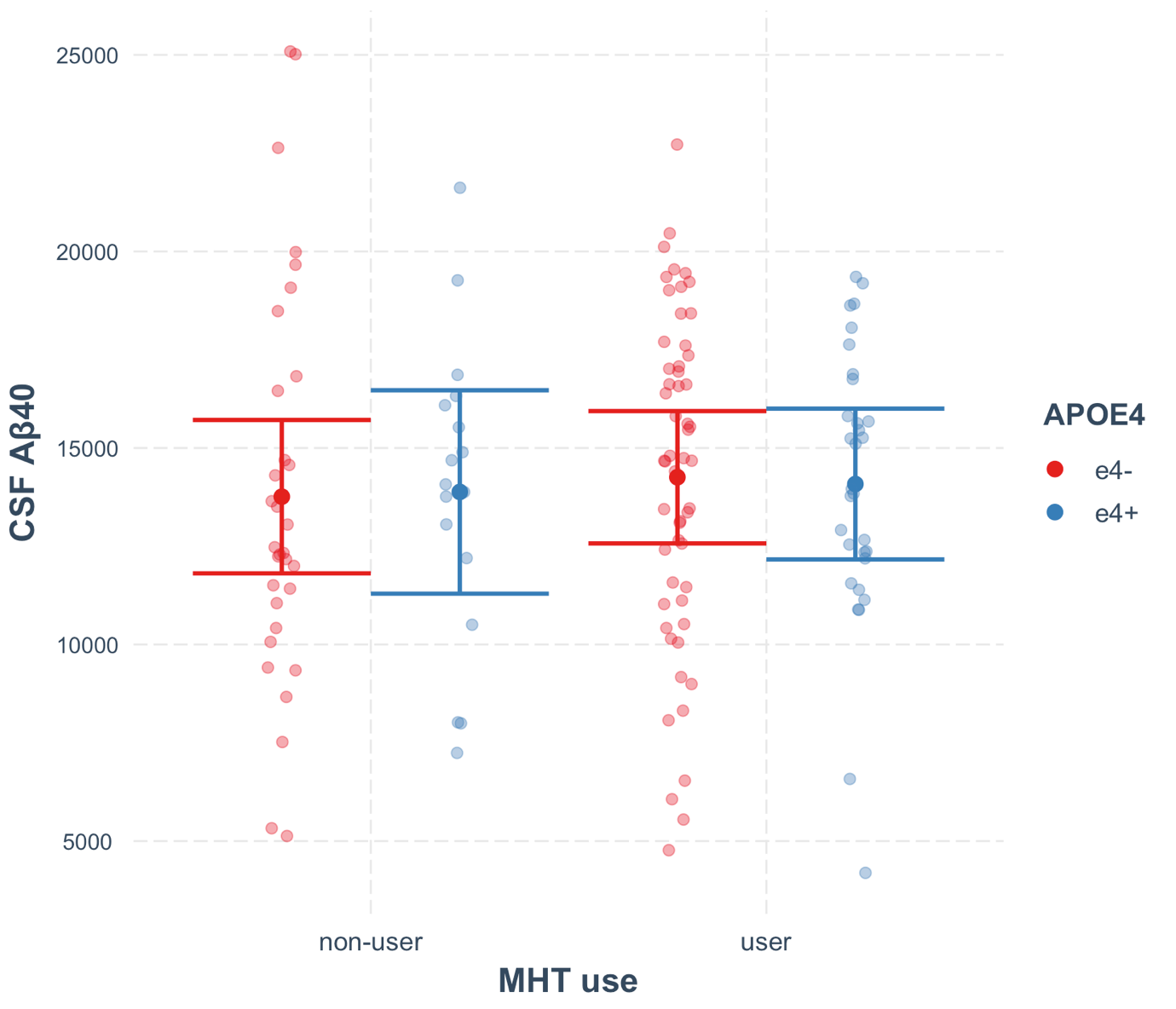
**

*Note:* Estimated marginal means and CIs of CSF Aβ40 levels in each group: e4-MHT- = 13199, CI [9747, 16652]; e4+MHT- = 13320, CI [9395, 17245]; e4-MHT+ = 13693, CI [10195, 17191]; e4+MHT+ = 13521, CI [9775, 17267]. Abbreviations: Aβ: amyloid-β; APOE4: apolipoprotein E ε4 allele, e4- are non-carriers, e4+ represent carriers; MHT: menopausal hormone therapy. *Post hoc* pairwise group comparisons: adjusted-*p* = .928, for all; unadjusted: ε4+MHT+ vs. ε4-MHT+, *p* = .863; ε4+MHT+ vs. ε4-MHT-, *p* = .779; ε4+MHT+ vs. ε4+MHT-, *p* = .887; ε4-MHT- vs. ε4+MHT-, *p* = .928; ε4-MHT- vs. ε4-MHT+, *p* = .631; ε4+MHT- vs. ε4-MHT+, *p* = .776).

**Figure S4.** Estimated marginal means of CSF levels of p-tau by MHT use and *APOE*4 carrier status, controlling for the covariates, with 95% confidence interval.

**
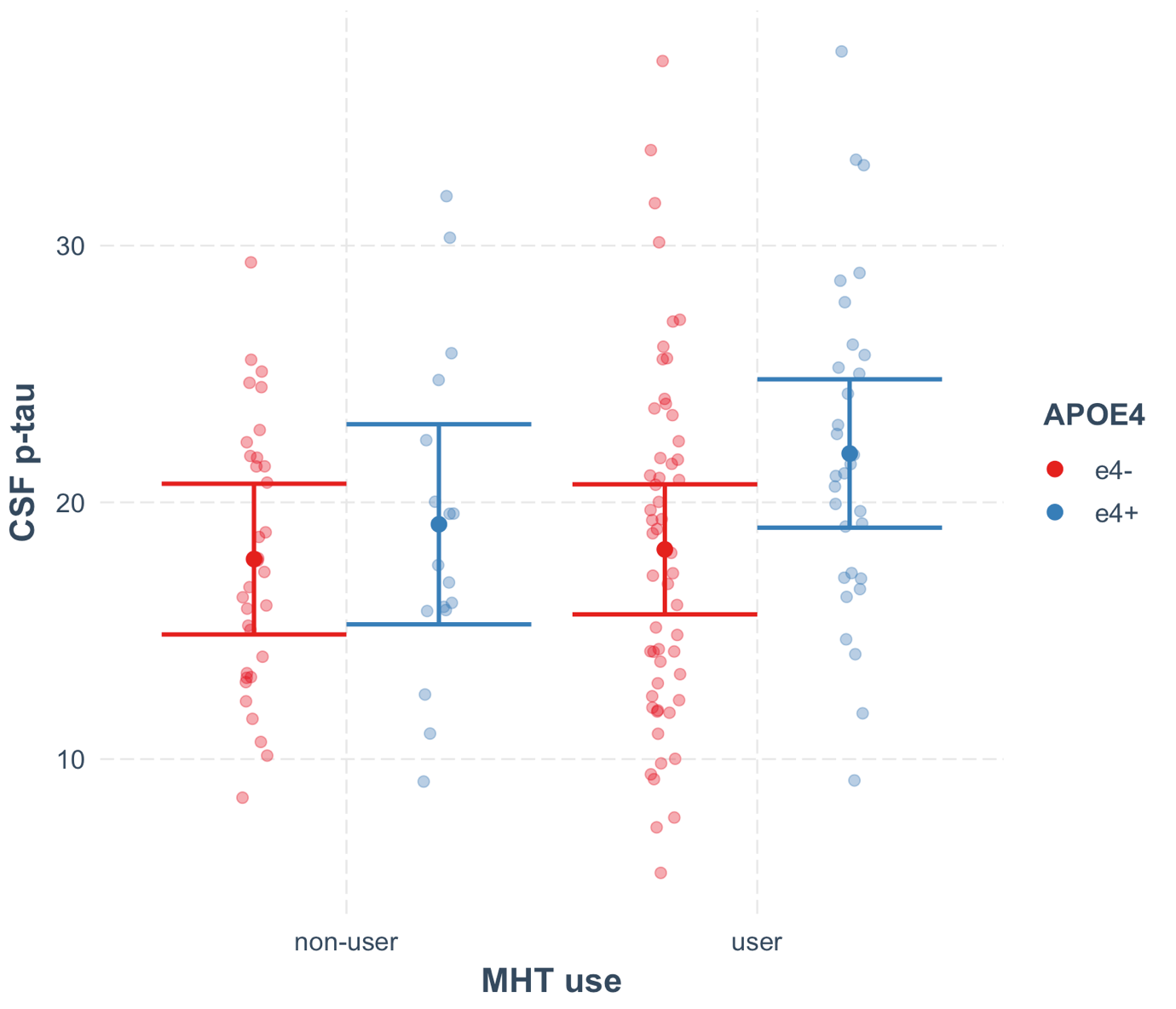
**

*Note:* Estimated marginal means and CIs of CSF p-tau levels in each group: e4-MHT- = 17.4, CI [12.2, 22.6]; e4+MHT- = 18.7, CI [12.8, 24.6]; e4-MHT+ = 17.8, CI [12.5, 23.0]; e4+MHT+ = 21.5, CI [15.8, 27.1]. Abbreviations: Aβ: amyloid-β; APOE4: apolipoprotein E ε4 allele, e4- are non-carriers, e4+ represent carriers; MHT: menopausal hormone therapy. *Post hoc* pairwise group comparisons: ε4+MHT+ vs. ε4-MHT+, adjusted-*p* = .056; ε4+MHT+ vs. ε4-MHT-, adjusted-*p* = .056; ε4+MHT+ vs. ε4+MHT-, adjusted-*p* = .396; ε4-MHT- vs. ε4+MHT-, adjusted-*p* = .746; ε4-MHT- vs. ε4-MHT+, adjusted-*p* = .807; ε4+MHT- vs. ε4-MHT+, adjusted-*p* = .746).

**Table S1.** Main effects and interaction models (APOE4 * MHT use) with (log-transformed) CSF Aβ42 as outcome.

|  | | |
| --- | --- | --- |
|  | Dependent variable: | |
|  |  | |
|  | CSF (log-transformed) Aβ42 | |
|  | Main Effects | Interaction |
|  | B (SE) | B (SE) |
|  | | |
| Constant | 2.905*** (0.043) | 2.878*** (0.045) |
| Age at LP | -0.005 (0.004) | -0.006 (0.004) |
| Education years | -0.004 (0.009) | -0.004 (0.009) |
| Elapsed time in years | 0.004 (0.003) | 0.005 (0.003) |
| Race(Black) | -0.186 (0.107) | -0.207 (0.106) |
| Race(Other) | -0.114 (0.207) | -0.084 (0.206) |
| Surgery(Yes) | -0.032 (0.038) | -0.046 (0.039) |
| Age at menarche | -0.006 (0.013) | -0.006 (0.013) |
| LIBRA(Moderate) | 0.048 (0.042) | 0.055 (0.042) |
| LIBRA(High) | 0.137** (0.047) | 0.135** (0.046) |
| APOE4(e4+) | -0.098** (0.037) | -0.003 (0.061) |
| MHT(user) | 0.007 (0.040) | 0.056 (0.047) |
| APOE4(e4+):MHT(user) |  | -0.149 (0.077) |
|  | | |
| Observations | 136 | 136 |
| R^2^ | 0.151 | 0.176 |
| Adjusted R^2^ | 0.076 | 0.096 |
| F Statistic | 2.002* (df = 11; 124) | 2.189* (df = 12; 123) |
|  | | |
| Note: Abbreviations: LP: lumbar puncture; Aβ: amyloid-β; APOE4: apolipoprotein E ε4 allele, e4+ represent carriers; MHT: menopausal hormone therapy; df = degrees of freedom. ^*^*p* < .05; ^**^*p* < .01; ^***^*p* < .001 | | |

**Table S2.** Main effects and interaction models (APOE4 * MHT use) with CSF Aβ40 as outcome.

|  | | |
| --- | --- | --- |
|  | Dependent variable: | |
|  |  | |
|  | CSF Aβ40 | |
|  | Main Effects | Interaction |
|  | B (SE) | B (SE) |
|  | | |
| Constant | 13,814.270*** (934.130) | 13,761.410*** (985.382) |
| Age at LP | 131.781 (93.661) | 130.469 (94.329) |
| Education years | 175.722 (192.440) | 174.945 (193.248) |
| Elapsed time in years | 66.235 (72.643) | 67.652 (73.378) |
| Race(Black) | -1,228.579 (2,299.658) | -1,269.497 (2,320.544) |
| Race(Other) | -4,281.737 (4,462.754) | -4,222.727 (4,493.010) |
| Surgery(Yes) | -193.865 (826.648) | -220.531 (843.811) |
| Age at menarche | 38.507 (278.844) | 38.879 (279.949) |
| LIBRA(Moderate) | 404.707 (902.366) | 417.566 (908.897) |
| LIBRA(High) | 3,723.473*** (1,005.305) | 3,718.310*** (1,009.691) |
| APOE4(e4+) | -67.879 (791.810) | 120.753 (1,340.548) |
| MHT(user) | 397.195 (858.189) | 494.192 (1,024.878) |
| APOE4(e4+):MHT(user) |  | -293.236 (1,678.010) |
|  | | |
| Observations | 136 | 136 |
| R^2^ | 0.169 | 0.169 |
| Adjusted R^2^ | 0.095 | 0.088 |
| F Statistic | 2.285* (df = 11; 124) | 2.081* (df = 12; 123) |
|  | | |
| Note: Abbreviations: LP: lumbar puncture; Aβ: amyloid-β; APOE4: apolipoprotein E ε4 allele, e4+ represent carriers; MHT: menopausal hormone therapy; df = degrees of freedom. ^*^*p* < .05; ^**^*p* < .01; ^***^*p* < .001 | | |

**Table S3.** Main effects and interaction models (APOE4 * MHT use) with CSF p-tau as outcome.

|  | | |
| --- | --- | --- |
|  | Dependent variable: | |
|  |  | |
|  | CSF p-tau | |
|  | Main Effects | Interaction |
|  | B (SE) | B (SE) |
|  | | |
| Constant | 17.361*** (1.412) | 17.791*** (1.484) |
| Age at LP | 0.325* (0.142) | 0.336* (0.142) |
| Education years | 0.520 (0.291) | 0.526 (0.291) |
| Elapsed time in years | 0.056 (0.110) | 0.044 (0.110) |
| Race(Black) | 4.655 (3.475) | 4.987 (3.494) |
| Race(Other) | -6.451 (6.744) | -6.930 (6.766) |
| Surgery(Yes) | -0.199 (1.249) | 0.018 (1.271) |
| Age at menarche | 0.484 (0.421) | 0.481 (0.422) |
| LIBRA(Moderate) | -1.304 (1.364) | -1.409 (1.369) |
| LIBRA(High) | 2.031 (1.519) | 2.073 (1.520) |
| APOE4(e4+) | 2.886* (1.196) | 1.354 (2.019) |
| MHT(user) | 1.165 (1.297) | 0.378 (1.543) |
| APOE4(e4+):MHT(user) |  | 2.381 (2.527) |
|  | | |
| Observations | 136 | 136 |
| R^2^ | 0.190 | 0.196 |
| Adjusted R^2^ | 0.118 | 0.117 |
| F Statistic | 2.646** (df = 11; 124) | 2.497** (df = 12; 123) |
|  | | |
| Note: Abbreviations: LP: lumbar puncture; Aβ: amyloid-β; APOE4: apolipoprotein E ε4 allele, e4+ represent carriers; MHT: menopausal hormone therapy; df = degrees of freedom. ^*^*p* < .05; ^**^*p* < .01; ^***^*p* < .001 | | |

**Table S4.** Main effects and interaction models (APOE4 * Age at MHT initiation) with CSF (log-transformed) p-tau/Aβ42 ratio as outcome.

|  | | |
| --- | --- | --- |
|  | Dependent variable: | |
|  |  | |
|  | CSF (log-transformed) p-tau/Aβ42 ratio | |
|  | Main Effects | Interaction |
|  | B (SE) | B (SE) |
|  | | |
| Constant | -2.065*** (0.527) | -2.607*** (0.533) |
| Age at LP | 0.020* (0.009) | 0.021* (0.008) |
| Education years | 0.030 (0.018) | 0.037* (0.017) |
| Elapsed time in years | -0.009 (0.007) | -0.008 (0.006) |
| Race(Black) | 0.462* (0.201) | 0.533** (0.191) |
| Surgery(Yes) | 0.107 (0.076) | 0.099 (0.071) |
| Age at menarche | 0.048 (0.025) | 0.047* (0.023) |
| LIBRA(Moderate) | -0.110 (0.070) | -0.129 (0.066) |
| LIBRA(High) | -0.064 (0.097) | -0.043 (0.092) |
| MHT Med (cEE) | 0.122 (0.129) | 0.132 (0.122) |
| MHT Med (cEE+Prog) | 0.177 (0.131) | 0.213 (0.124) |
| MHT Med (Oestrog+Prog) | 0.125 (0.124) | 0.171 (0.118) |
| MHT Form (Cream) | -0.079 (0.124) | -0.079 (0.117) |
| MHT Form (Ring) | 0.127 (0.184) | 0.100 (0.173) |
| MHT Form (Combined) | 0.041 (0.104) | 0.036 (0.098) |
| MHT Duration | -0.008 (0.007) | -0.012 (0.007) |
| APOE4(e4+) | 0.325*** (0.065) | 1.808** (0.533) |
| Age at MHT initiation | -0.008 (0.009) | 0.003 (0.009) |
| APOE4(e4+):Age at MHT |  | -0.030** (0.011) |
|  | | |
| Observations | 73 | 73 |
| R^2^ | 0.417 | 0.491 |
| Adjusted R^2^ | 0.237 | 0.321 |
| F Statistic | 2.315** (df = 17; 55) | 2.895** (df = 18; 54) |
|  | | |
| Note: Abbreviations: LP: lumbar puncture; Aβ: amyloid-β; APOE4: apolipoprotein E ε4 allele, e4+ represent carriers; MHT: menopausal hormone therapy; df = degrees of freedom. ^*^*p* < .05; ^**^*p* < .01; ^***^*p* < .001 | | |

**Table S5.** Main effects and interaction models (APOE4 * Age at MHT initiation) with CSF Aβ42/40 ratio as outcome.

|  | | |
| --- | --- | --- |
|  | Dependent variable: | |
|  |  | |
|  | CSF Aβ42/40 ratio | |
|  | Main Effects | Interaction |
|  | B (SE) | B (SE) |
|  | | |
| Constant | 0.060 (0.038) | 0.096* (0.038) |
| Age at LP | -0.002** (0.001) | -0.002** (0.001) |
| Education years | -0.002 (0.001) | -0.002 (0.001) |
| Elapsed time in years | 0.001 (0.0005) | 0.001 (0.0004) |
| Race(Black) | -0.025 (0.014) | -0.030* (0.014) |
| Surgery(Yes) | -0.008 (0.005) | -0.007 (0.005) |
| Age at menarche | -0.003 (0.002) | -0.003 (0.002) |
| LIBRA(Moderate) | 0.008 (0.005) | 0.009 (0.005) |
| LIBRA(High) | 0.002 (0.007) | 0.0005 (0.007) |
| MHT Med (cEE) | -0.016 (0.009) | -0.016 (0.009) |
| MHT Med (cEE+Prog) | -0.013 (0.009) | -0.016 (0.009) |
| MHT Med (Oestrog+Prog) | -0.014 (0.009) | -0.017 (0.009) |
| MHT Form (Cream) | 0.007 (0.009) | 0.007 (0.008) |
| MHT Form (Ring) | -0.021 (0.013) | -0.019 (0.013) |
| MHT Form (Combined) | -0.006 (0.007) | -0.006 (0.007) |
| MHT Duration | 0.001 (0.001) | 0.001* (0.001) |
| APOE4(e4+) | -0.023*** (0.005) | -0.121** (0.038) |
| Age at MHT initiation | 0.001 (0.001) | 0.0004 (0.001) |
| APOE4(e4+):Age at MHT |  | 0.002* (0.001) |
|  | | |
| Observations | 73 | 73 |
| R^2^ | 0.430 | 0.491 |
| Adjusted R^2^ | 0.254 | 0.322 |
| F Statistic | 2.439** (df = 17; 55) | 2.898** (df = 18; 54) |
|  | | |
| Note: Abbreviations: LP: lumbar puncture; Aβ: amyloid-β; APOE4: apolipoprotein E ε4 allele, e4+ represent carriers; MHT: menopausal hormone therapy; df = degrees of freedom. ^*^*p* < .05; ^**^*p* < .01; ^***^*p* < .001 | | |
